# 2,2′-Biquinoline Modified Expanded Graphite Electrode for the Detection of Cuprous Ions in Electrolytic Copper Foil Electrolyte

**DOI:** 10.3390/ma19030586

**Published:** 2026-02-03

**Authors:** Zhiyao Ming, Wenchang Wang, Ding Jiang, Pengju Wang, Yufa Sun, Qihu Wu, Zhidong Chen

**Affiliations:** School of Materials Science and Engineering, Changzhou University, Changzhou 213164, China; b23010805010@smail.cczu.edu.cn (Z.M.); jiangding0323@163.com (D.J.); pengjuwangcz@163.com (P.W.); syufa1013@163.com (Y.S.); wuqihu1698@163.com (Q.W.)

**Keywords:** Cu^+^, 2,2′-Biquinoline, expanded graphite, specific adsorption, selective determination

## Abstract

**Highlights:**

**What are the main findings?**

- A 2,2′-biquinoline (BIQ)-modified expanded graphite (EG) electrode electrochemical sensor was fabricated for the selective determination of Cu^+^ in copper sulfate electrolyte, and differential pulse voltammetry (DPV) was adopted for the electrochemical detection of Cu^+^ with high sensitivity, excellent selectivity, a low detection limit and a rapid response.
- BIQ’s specific coordination with Cu^+^ enables the modified sensor to realize rapid and effective quantification of Cu^+^; the BIQ-modified EG electrode exhibits outstanding selectivity for Cu^+^, with a Cu^+^ recovery rate of 101.00–105.00% even in the presence of 10,000-fold excess Cu^2+^ and a relative standard deviation (RSD) of less than 2%.

**What are the implications of the main findings?**

- The developed BIQ-modified EG electrode sensor provides a novel, accurate and efficient analytical method for the selective detection of trace Cu^+^ in the complex electrolytic copper foil electrolyte system with high-concentration Cu^2+^ interference.
- Precise and reliable quantification of Cu^+^ in copper sulfate electrolyte via this sensor can support the rational regulation of Cu^+^ content in the electrolyte, thereby facilitating the optimization of the microstructure and performance of electrolytic copper foil.

**Abstract:**

The coexistence of Cu in copper sulfate electrolyte significantly affects the microstructure and performance of the copper foil. So far, there has been little quantitative analysis of Cu^+^ in the electrolyte during the copper foil production process. This paper fabricated a 2,2′-Biquinoline (BIQ) modified expanded graphite (EG) electrode electrochemical sensor for the selective determination of Cu^+^. EG, with its large specific surface area and excellent adsorption and electrochemical properties, significantly enhances analytical sensitivity. Additionally, BIQ’s specific coordination with Cu^+^ improves the sensor’s rapid and effective quantification of Cu^+^ in the electrolytic copper foil electrolyte. The linear equation of this sensor is I = 0.03769 + 0.29997 × c (R^2^ = 0.9989), with a detection limit of 8 μg/L (S/N = 3). The BIQ-modified EG electrode has good selectivity for Cu^+^, with a recovery rate for cuprous ions of 101.00% to 105.00% under the coexistence of 10,000 times Cu^2+^, and an RSD of less than 2%. This sensor’s efficient, sensitive, and selective detection of Cu^+^ can be an effective method to improve the quality of electrolytic copper foil products.

## 1. Introduction

In the rapidly evolving era of 5G technology, high-quality copper foil is essential in the fabrication of semiconductor devices, where it functions not only as a substrate for circuit boards, a heat dissipation layer for devices, and electrode material, but also enhances the overall performance of semiconductor devices by providing excellent conductivity, stable electrical properties, and improved heat dissipation. However, during the preparation of copper foil, the presence of a certain amount of Cu^+^ in the solution is often due to incomplete electrochemical reactions or impurities in the electrolyte. Thus, obtaining high-quality copper foil requires strict control of Cu^+^ in the electrolytic solution [1,2]. Cu^+^ coexistence in the electrolyte may cause defects like burrs, pinholes, and copper nodules on the copper foil, adversely affecting its microstructure and physical properties [3,4,5]. On the other hand, Cu^+^ can react with additives such as polyethylene glycol [6], bis(3-sulfopropyl) disulfide [7], chloride ions [8], etc., forming stable complexes, leading to the failure of the additives and affecting the performance of copper foil products. Therefore, real-time monitoring and quantitative analysis of Cu^+^ in the electrolyte are very important for obtaining high-quality copper foil.

Recently, the main analytical methods for detecting Cu^+^ include spectrophotometry [9], capillary electrophoresis [10], and fluorescence methods [11,12,13]. Current research primarily focuses on the qualitative detection of Cu^+^ in copper plating to study its behavior and impact on plating quality [14,15,16]. On the other hand, the complexity of the electrolytic system and numerous divalent copper ions complicate the selective determination of Cu^+^, affecting analytical accuracy and posing challenges.

The electrochemical detection of Cu^+^ using differential pulse voltammetry (DPV) offers high sensitivity, excellent selectivity, low detection limits, and rapid response. However, conventional electrode materials often suffer from insufficient sensitivity, poor selectivity, and high detection limits. Therefore, there is a need to explore high-performance electrode materials.

Expanded graphite (EG), as a material with great development prospects, has many advantages such as low cost, easy availability, and environmental friendliness, and is widely used as an abrasion-resistant material [17], adsorbent [18], and conductive material [19,20], among others. It has been used in the analytical field as a base electrode for modified electrochemical sensors [21,22]. Finding effective target sites on the electrode surface is key to the selective determination by electrochemical sensors. 2,2′-Biquinoline (BIQ) is known as a sub-copper reagent, reacting with Cu^+^ to form a purple complex, serving as a highly effective chromogenic agent for Cu^+^ [23,24,25]. This electrode offers a novel and effective method for monitoring Cu^+^ in copper sulfate solutions.

This work prepared a BIQ-modified EG paste electrode for the selective determination of Cu^+^. Simultaneously, this electrode demonstrates good selectivity for Cu^+^, providing a novel and effective method for monitoring Cu^+^ in copper sulfate solutions.

## 2. Experimental

### 2.1. Chemicals and Apparatus

BIQ, hydrochloric acid, hydroxylamine hydrochloride, sodium sulfate, copper sulfate, zinc nitrate, calcium chloride, nickel chloride, potassium chloride, etc., were purchased from Sinopharm Chemical Reagent Co., Ltd., Shanghai, China. Natural flake graphite (FG) (C, 99%, 50 mesh) was produced by Qingdao Haida Graphite Co., Ltd., Qingdao, China. Solid paraffin was purchased from Yixing Reagent Factory No. 2, Yixing, China. All chemicals were of analytical reagent (AR) grade and were used without further purification.

A certain amount of CuSO_4_·5H_2_O was dissolved in deionized water to prepare a 100 mg/L Cu^2+^ standard solution. After the solution was subjected to continuous nitrogen bubbling for 15–20 min, it was mixed with hydroxylamine hydrochloride at a 1.5-fold stoichiometric ratio in a specified volume, and 0.5 mol/L H_2_SO_4_ was added to maintain an acidic environment. Cu^+^ standard solutions with a concentration range of 0.05 mg/L to 1.0 mg/L were generated via a redox reaction, and the excess hydroxylamine hydrochloride ensured the complete reduction of Cu^2+^ to Cu^+^. We also referred to the standard colorimetric method reported in Ref. [26] and used neocuproine hydrochloride monohydrate to verify the Cu^+^ concentration.

The electrochemical experiments were performed with the CHI 410A electrochemical analyzing system (model CHI410A, Chenhua Instrument Co., Ltd., Shanghai, China) with a conventional three-electrode cell. UV–visible absorption spectra were measured at room temperature using a UVmini-1240 spectrophotometer (Shimadzu, Kyoto, Japan). Scanning electron microscopes (SEM) observation of the EG and flake graphite (FG) were carried out with a JSM-6360LA SEM instrument (JEOL Ltd., Tokyo, Japan). The transmission electron microscope (TEM) images were obtained using an HT-7800 (Hitachi, Tokyo, Japan) transmission electron microscope. N_2_ adsorption/desorption isotherms were carried out at 77 K using an STA 449F3 (Netzsch, Selb, Germany) adsorption instrument. FT-IR analysis was performed on Nicolet iN10 Fourier transform microscopy infrared spectrometer (Thermo Fisher Scientific, Shanghai, China) and the X-ray diffraction (XRD) was recorded on the Max-2000 (Rigaku Co., Ltd., Tokyo, Japan).

### 2.2. Preparation of Modified Electrode

Preparation of EG:FG, potassium permanganate, nitric acid, and phosphoric acid were mixed at room temperature in a ratio of 1 (g):0.25 (g):4 (mL):12 (mL). After reacting for 100 min, the mixture was filtered and washed with water until neutral, dried at 80 °C, and then expanded in a muffle furnace at 900 °C for 60 s to obtain EG.

Electrode preparation: First, dissolve BIQ in 3 mL of anhydrous ethanol and heated to 60 °C. Then, 0.03 g of EG was added to uniformly adsorb the BIQ. Afterwards, 0.02 g of solid paraffin was heated and dissolved, then was mixed with the BIQ-adsorbed EG, and stirred for 5 min. Finally, fill the mixture into one end of a glass tube (5 mm diameter) and insert a copper wire (2 mm diameter) through the other end to establish electrical contact. Polish the electrode surface on weighing paper to smooth it and produce a BIQ-modified EG electrode. Using FG instead of the EG mentioned above can produce a BIQ-modified carbon electrode.

### 2.3. Preparation of DPV

The BIQ-modified EG paste electrode was immersed for 10 min in various Cu^+^ solutions and a 31.85 g/L Cu^2+^ solution for enrichment. Subsequently, the electrodes were transferred to a 0.1 M Na_2_SO_4_ solution (pH 7.0), and differential pulse voltammetry (DPV) was employed. The three-electrode system consisted of a BIQ-modified EG electrode as the working electrode, a platinum sheet as the counter electrode, and an Ag/AgCl electrode as the reference electrode. The test parameters were set as follows: potential increment of 10 mV, pulse amplitude of 0.1 V, pulse width of 1.0 s, and pulse period of 0.2 s.

## 3. Results and Discussion

### 3.1. Characterization of Electrode Materials

**Figure 1:**
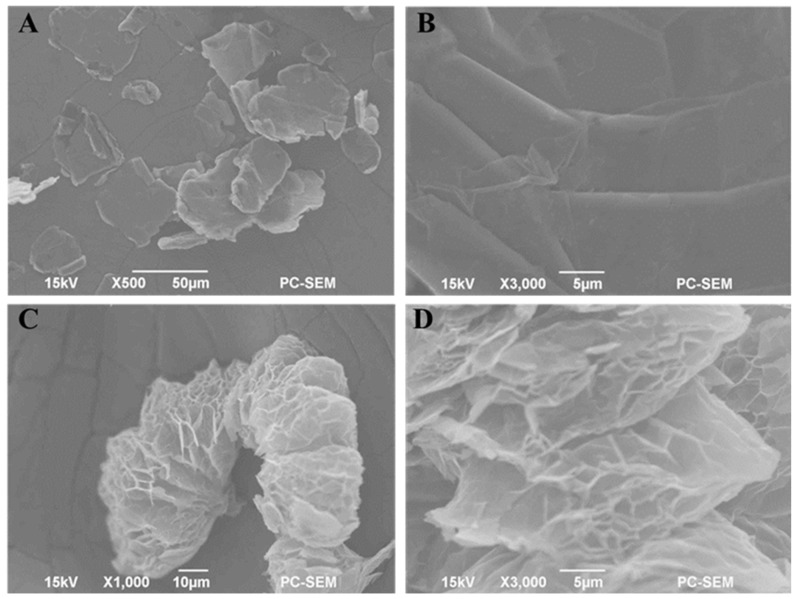
SEM micrographs of FG (**A**,**B**) and EG (**C**,**D**).

Scanning Electron Microscopy (SEM) characterized the surface morphology of FG and EG. Figure 1A,B show that FG has a lamellar structure with layered stacks. Gaps of varying degrees are present between the layers. EG is formed by high-temperature expansion in a muffle furnace after mixed acids like phosphoric and nitric acids penetrate the gaps in FG layers and undergo secondary oxidation with potassium permanganate. Figure 1C,D depict EG as having a loose, worm-like structure with numerous micropores of varying sizes. The increased specific surface area of this structure enhances BIQ adsorption.

**Figure 2:**
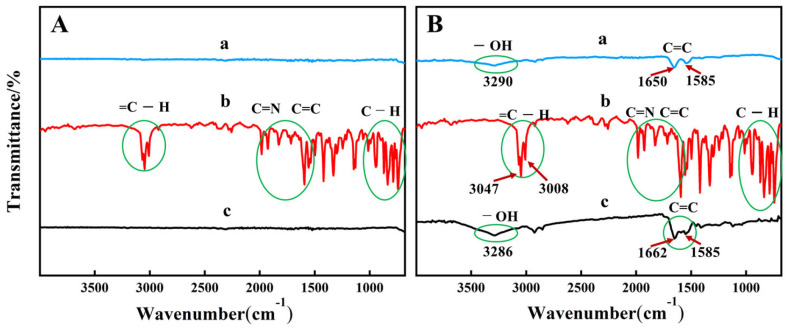
FT-IR spectra of FG (**A**) and EG (**B**) before and after adsorption of BIQ. (**A**): (a) FG; (b) BIQ; (c) FG after adsorption. (**B**): (a) EG; (b) BIQ; (c) EG after adsorption of BIQ.

Weigh 0.05 g of EG and add it to 20 mL of a 5 mg/L BIQ n-butanol solution, stir for 30 min, filter, analyze the filtrate with UV-visible spectroscopy, and analyze the residue with FT-IR spectroscopy. Figure 2A,B showed the infrared spectra of FG and EG before and after adsorption of BIQ, respectively. From Figure 2A, the b curve represents the infrared spectrum of BIQ. BIQ shows C-H out-of-plane bending vibrations in the range of 680~1200 cm^−1^. The ring skeletal vibration region is 1200~1600 cm^−1^. The stretching vibration region for C=C and C=N is located at 1600~2000 cm^−1^. The =C-H stretching vibration region is at 2900~3100 cm^−1^. The infrared spectrum curve (c curve) of FG after adsorbing BIQ does not show the characteristic peaks of BIQ. This indicates that FG does not have the ability to adsorb BIQ. EG residues adsorbed with BIQ and solid EG materials after BIQ adsorption exhibit consistent peak positions in the FTIR spectra (Figure S5).

**Figure 3:**
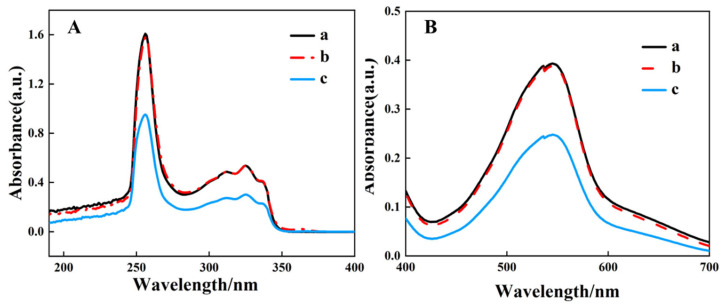
(**A**): UV-visible absorption spectra of BIQ n-butanol solution (a) and the filtrates after stirring and filtering with equal amounts of FG (b) and expanded EG (c). (**B**): UV-visible absorption spectra of the organic phase after extraction (a) and the filtrates after stirring and filtering with FG (b) and EG (c).

Add 20 mL of a 0.2 g/L BIQ solution and 5 mL of a 12.5 mg/L Cu^+^ solution to a separatory funnel and carry out the extraction experiment to obtain the organic phase solution containing the BIQ and Cu^+^ complex. Take 20 mL of the organic phase solution, add 0.03 g of EG, stir for 30 min, filter, and collect the filtrate. Figure 3A presents the UV-visible absorption spectra for a 5 mg/L BIQ solution and filtrates obtained after stirring and filtering with equal amounts of FG and EG. Curve b shows the absorption spectrum of the filtrate with added FG, where 0.05 g of FG decreased the BIQ n-butanol solution’s UV absorption by 0.0306 AU at 256 nm and 0.0004 a.u. at 325 nm. Curve c shows the absorption spectrum of the filtrate with EG; adding EG significantly reduced the BIQ-butanol solution’s absorption to 0.9517 a.u. at 256 nm and 0.3013 a.u. at 325 nm. This comparison indicates that EG has a higher adsorption capacity for BIQ than FG. Based on this, the adsorption performance expanded and FG for the BIQ and Cu^+^ complex was tested, with the results shown in Figure 3B. Curve a displays the UV-visible spectrum of the organic phase post-extraction, Curve b for the filtrate with FG, and Curve c for the filtrate with EG. Curve a exhibits a peak at 545 nm, attributed to the [Cu(BIQ)_2]^2+^ complex, confirming its presence. Curve b indicates that adding 0.03 g of FG did not affect the absorbance intensity of the filtrate. In contrast, Curve c shows that adding EG reduced the absorbance at 545 nm from 0.40 a.u. to 0.25 a.u., demonstrating EG’s superior adsorption capacity for the BIQ-Cu^+^ complex.

### 3.2. Electrochemical Characterization

**Figure 4:**
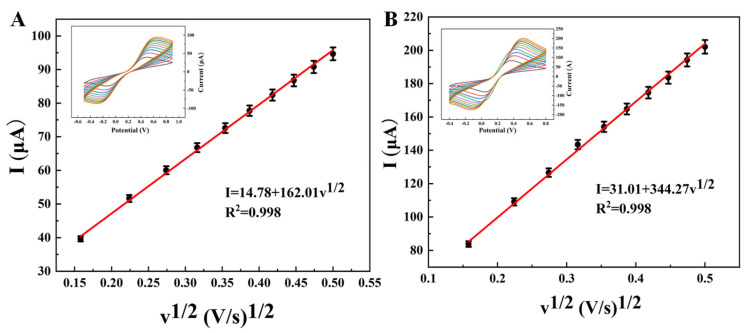
CV responses and the corresponding linear relationships of (**A**) FG and (**B**) EG electrodes modified with 5 mg of BIQ. Measurements were performed in a 5.0 mmol·L^−1^ K_3_[Fe(CN)_6_] solution at scan rates ranging from 25 to 250 mV·s^−1^, (25 mV·s^−1^ increments), with a corresponding enhancement of the redox peaks on the curves. Figure 5 shows the differential pulse voltammograms of a BIQ-modified EG electrode as the working electrode after 10 min of enrichment in solutions containing 1 mg/L Cu^+^, 31.25 g/L Cu^2+^, and their coexistence, tested in 0.1 M Na_2_SO_4_ solution (pH = 7). When the enrichment solution only contained Cu^+^, its DPV Curve (Curve b) exhibited an oxidation peak at 0.55 V, with a peak current of 0.3311 μA. However, when the enrichment solution only contained Cu^2+^, its DPV Curve (Curve a) exhibited a peak current of merely 0.049 μ, much less than that of Curve b, indicating that the interference from divalent copper ions was weak. When both were present, the change in the oxidation peak current (Curve c) compared to the Cu^+^ solution was 2.7% (<5%), indicating the feasibility of detecting Cu^+^ using differential pulse voltammetry with a BIQ-modified EG paste electrode.

We selected K_3_[Fe(CN)_6_] as the standard redox probe, and fabricated FG and EG electrodes adsorbed with 5 mg BIQ, respectively. Cyclic voltammetry (CV) tests were performed to obtain the electrochemical response curves of the two electrodes at different scan rates. Scan rates increased from 25 to 250 mV·s^−1^. The ECSA values of the electrodes were calculated based on the slope of the linear fitting between peak current and the square root of scan rate, combined with the Randles-Sevcik equation. The specific results are shown in Figure 4: the ECSA of the FG electrode is 46.22 cm^2^, while that of the EG electrode is 98.93 cm^2^, meaning the ECSA of the EG electrode is 2.14 times that of the FG electrode.

This quantitative result intuitively and strongly confirms that, compared with FG, EG formed by intercalation and expansion treatment has a more developed porous structure. Its larger electrochemical active surface area enables the loading of more BIQ to provide sufficient active sites for the specific coordination of Cu^+^.

**Figure 5:**
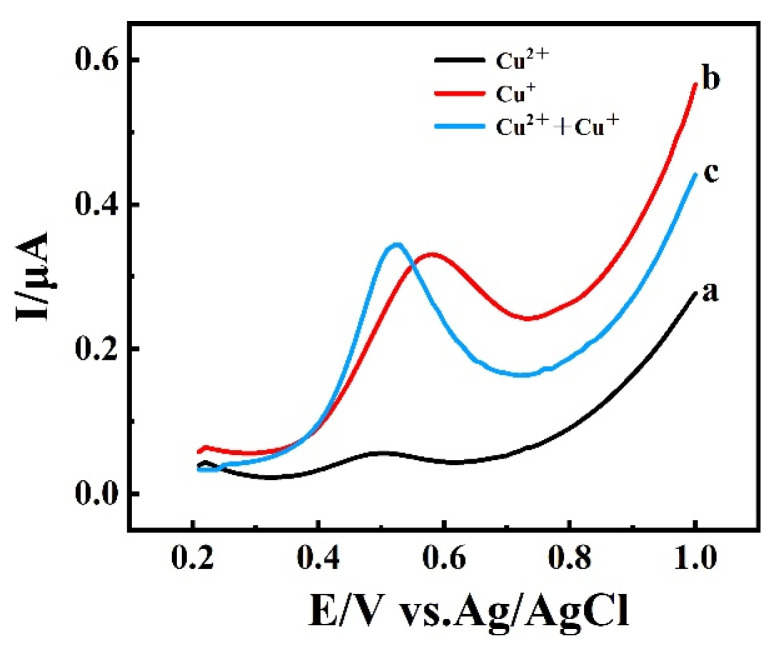
DPV curves of BIQ-modified EG electrode after enrichment in solutions with different components: (a) 31.85 g/L Cu^2+^, (b) 1 mg/L Cu^+^, (c) 31.85 g/L Cu^2+^, and 1 mg/L Cu^+^.

### 3.3. Optimization of Detection Conditions

**Figure 6:**
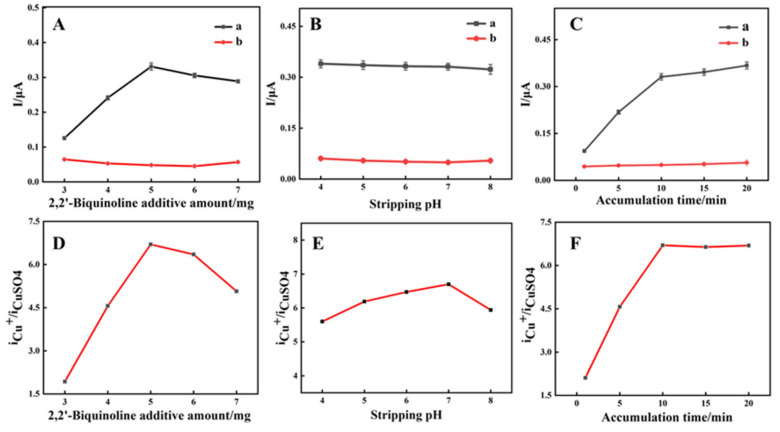
(**A**–**C**): The effects of BIQ content, leaching solution pH, and enrichment time on the DPV curve’s oxidation peak current: (a) 1 mg/L Cu^+^, (b) 31.85 g/L Cu^2+^. (**D**–**F**) each correspond to the ratio of oxidation peak currents in solutions containing Cu^+^ and Cu^2+^ for (**A**–**C**), respectively.

To optimize sensitivity, the effect of varying BIQ amounts (3, 4, 5, 6, and 7 mg) on Cu^+^ detection was examined, with results displayed in Figure 6. Figure 6A shows that the Cu^+^ oxidation peak current initially increases, then decreases with increasing BIQ amounts from 3 mg to 7 mg. The peak oxidation current is maximum at 5 mg. The rise in oxidation peak current is due to more Cu^+^ complexing as the BIQ amount increases, peaking at 0.3311 μA with 5 mg of BIQ. Further increases in BIQ beyond 5 mg raise electrode surface resistance, reducing peak current. The slight decrease in Cu^2+^ oxidation peak current suggests minimal BIQ influence. Figure 6D illustrates the oxidation peak current ratio of Cu^+^ to Cu^2+^, highest at 6.7 with 5 mg BIQ, indicating optimal BIQ addition.

Figure 6B examines the oxidation peak currents of Cu^+^ and Cu^2+^ against the pH of leaching solutions, with Curve a for Cu^+^ and Curve b for Cu^2+^. The Cu+ oxidation peak current decreased from 0.33980 μA to 0.32310 μA and the Cu^2+^ from 0.06066 μA to 0.05437 μA as the leachate pH increased from 4 to 8. This indicates minimal pH effect on the electrode’s selectivity for Cu^+^. Figure 6E shows the oxidation peak current ratio of Cu^+^ to Cu^2+^ peaks at 6.7 when the leach solution pH is 7.

Figure 6C demonstrates the significant impact of enrichment time on the electrode’s efficiency, where the Cu^+^ oxidation peak current increased from 0.09411 μA to 0.3311 μA, with enrichment time rising from 1 to 10 min. Beyond 10 min, the Cu^+^ peak current increment was just 0.0362 μA, likely due to adsorption saturation on the electrode. Figure 6F, Curve a, displays the Cu^2+^ oxidation peak current ratio over enrichment time. Hence, a 10 min enrichment time is selected to maximize peak current ratio and minimize analysis time.

### 3.4. Determination of Analytical Properties

**Figure 7:**
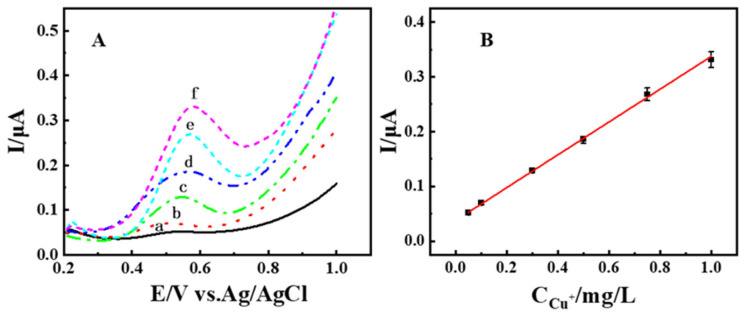
(**A**) DPV curves for solutions with different concentrations of Cu^+^: (a) 0.05 mg/L, (b) 0.1 mg/L, (c) 0.3 mg/L, (d) 0.5 mg/L, (e) 0.75 mg/L, (f) 1 mg/L (**B**) The related linear regression curve, with the linear relationship given by I = 0.03769 + 0.29997 × c (R^2^ = 0.99).

DPV testing was performed in standard solutions with different concentrations of Cu^+^ (0.05 mg/L to 1 mg/L), with an addition of 5 mg of BIQ, solution pH of 7, and an enrichment time of 10 min, with the final results displayed in Figure 7A. From Figure 6A, it can be seen that, as the concentration of Cu^+^ increases, the oxidation peak current of the electrode gradually increases. Within the range of 0.05 mg/L to 1 mg/L, the oxidation peak current shows a good linear relationship with Cu^+^ concentration (Figure 7B), the linear equation is I = 0.03769 + 0.29997 × c (R^2^ = 0.9989), and a detection limit of 8 μg/L (S/N = 3). The results indicate that the BIQ-modified EG electrode has a good linear range and detection limit for Cu^+^ response.

### 3.5. Anti-Interference Detection

**Figure 8:**
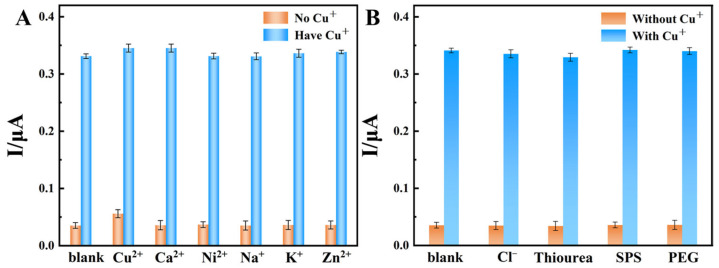
The effect of interfering factors on the detection of Cu^+^. (**A**) From left to right, the soaking solutions are deionized water, 1 mg/L Cu^+^, 80 g/L CuSO_4_, 80 g/L CuSO_4_ + 1 mg/L Cu^+^, 10 g/L CaCl_2_, 10 g/L CaCl_2_ + 1 mg/L Cu^+^, 10 g/L NiCl_2_, 10 g/L NiCl_2_ + 1 mg/L Cu^+^, 10 g/L Na_2_SO_4_, 10 g/L Na_2_SO_4_ + 1 mg/L Cu^+^, 10 g/L KCl, 10 g/L KCl + 1 mg/L Cu^+^, 10 g/L ZnNO_3_, and 10 g/L ZnNO_3_ + 1 mg/L Cu^+^; (**B**) From left to right, the soaking solutions are deionized water, 1 mg/L Cu^+^, 10 mg/L thiourea, 10 mg/L thiourea + 1 mg/L Cu^+^, 50 mg/L Cl^−^, 50 mg/L Cl^−^ + 1 mg/L Cu^+^, 10 mg/L SPS, 10 mg/L SPS + 1 mg/L Cu^+^, 50 mg/L PEG, 50 mg/L PEG + 1 mg/L Cu^+^.

The experiment investigated the influence of different coexisting ions on the detection of Cu^+^. Figure 8A shows the influence of coexisting interfering ions such as Zn^2+^, Ni^2+^, and Ca^2+^ on the detection of Cu^+^. The concentration of each coexisting ion was 10 g/L, while the concentration of Cu^+^ was 1 mg/LAs can be seen from the figure, the concentration of coexisting ions was 10,000 times that of Cu^+^, but this did not significantly affect the detection of Cu^+^. In Figure 8B, 10 mg/L thiourea only reduces the response peak current of the sensor to Cu^+^ by 3.4%, while the peak current change rates are all less than 2% in the presence of additives such as PEG (50 mg/L), SPS (10 mg/L), and Cl^−^ (50 mg/L). The above change amplitudes are all below 5%, indicating that these substances have no obvious interference on the detection of Cu^+^. This phenomenon is mainly attributed to the dual guarantees of thermodynamic advantage and structural stability of BIQ, our Cu^+^ capture agent: 2,2′-biquinoline (BIQ) forms a stable 1:2 complex [Cu(BIQ)_2_]^+^ [27] with Cu^+^, and possesses a relatively high stability constant. These data indicate that the prepared modified electrode can specifically detect Cu^+^ in solution.

### 3.6. Stability and Reproducibility

**Figure 9:**
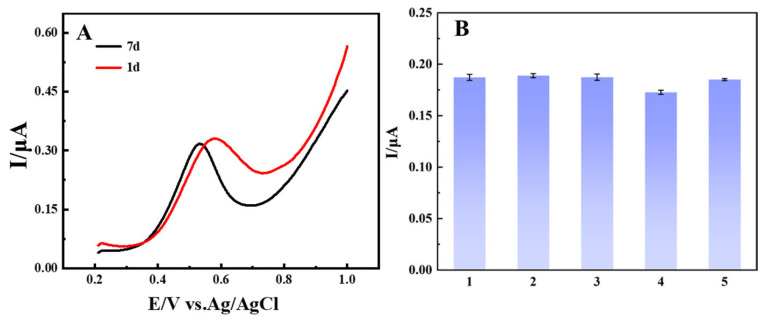
(**A**) The DPV detection curves of the newly made and 7-day-old electrodes (**B**) The peak oxidation current obtained from five repetitively prepared modified electrodes.

Under optimal experimental conditions, the stability of the newly made BIQ-modified EG electrode was evaluated by testing the DPV curves before and after 7 days of storage in a 1 mg/L Cu^+^ solution, with 10 min of enrichment. As shown in Figure 9A, the initial oxidation peak current of the electrode was 0.3311 μA, which changed to 0.3173 μA after being left at room temperature for 7 days, with a change in the measured DPV curve’s oxidation peak current of 4.1%, indicating good stability of the electrode.

Five electrodes were repeatedly prepared under optimal conditions, and each was tested with DPV after 10 min of enrichment in a 1 mg/L Cu^+^ standard solution to evaluate the reproducibility of the BIQ-modified EG paste electrode. The results, shown in Figure 9B, indicate that the peak currents for the five electrodes were 0.1872 μA, 0.1889 μA, 0.1874 μA, 0.1727 μA, and 0.1851 μA, respectively, with a relative standard deviation of 3.58%, demonstrating the good reproducibility of the electrodes prepared by this method.

### 3.7. Sample Analysis

Under optimal conditions, to verify the practical performance of the proposed BIQ-modified EG electrode, the recovery rate of Cu^+^ in the electrolyte was tested using spectrophotometry as a reference method, to evaluate the detection capability and accuracy of the electrode for actual samples. Prior to the test, the actual electrolyte was diluted at a volume ratio of 1:1 to obtain the test solution, so as to avoid the high-concentration Cu^2+^ matrix from affecting the stability of the detection signal. The experimental results are shown in Table 1. As shown in the table, the recovery rate of Cu^+^ in the copper solution ranged from 101.21% to 104.48%, with an RSD (Relative Standard Deviation) of less than 2%. The recovery rate and relative standard deviation both met expectations, indicating that the method is accurate and reliable. The developed BIQ-modified EG electrode can be used to determine Cu^+^ in electrolytic copper foil solutions.

## 4. Discussion

The interaction between BIQ and the EG surface is governed by specific π-π stacking rather than simple physical adsorption, and its high stability arises from the combined effects of molecular structure compatibility and interfacial synergistic effects. The molecular structure of BIQ provides an inherent basis for π-π stacking: as a planar conjugated molecule containing two quinoline rings, its conjugated system size (approximately 1.2 nm × 0.4 nm) exhibits good compatibility with the lattice structure of the graphene basal plane on the EG surface (lattice constant of 0.246 nm), enabling the formation of an “AB-type parallel stacking” mode [27]. This oriented arrangement can maximize the spatial overlap of π electron clouds, and the lone pair electrons of nitrogen atoms on the quinoline rings of BIQ can form additional electrostatic interactions with the delocalized π electrons of the graphene basal plane, further enhancing the interfacial binding.

Björk et al. [28] confirmed, through van der Waals density functional (vdW-DF) calculations and temperature-programmed desorption (TPD) experiments, that the π-π stacking interaction between aromatic conjugated molecules and graphene is centered on long-range dispersion forces, with short-range superimposed electrostatic contributions. Its single-atom binding energy reaches 49.2~80.1 meV, corresponding to a macroscopic adsorption energy of −38~−64 kJ/mol, which is much higher than the van der Waals force of physical adsorption, confirming the stability advantage of this interaction from the energy perspective.

At the structural level, the study by Chen et al. [29] further supports that the lattice-matched commensurate interface exhibits low interfacial resistance (<3 Ω) and high structural stability due to sufficient π electron cloud overlap (the overlap degree is 40% higher than that of the mismatched interface), and the compatibility between EG and BIQ exactly meets this interfacial characteristic. Ultimately, the prepared electrode possesses high selectivity, high reproducibility, and excellent electrochemical response performance.

## 5. Conclusions

This article presents a new type of electrochemical sensor for the selective determination of Cu^+^ in electrolytic copper foil solutions, based on the excellent conductivity of EG and its special adsorption capacity for BIQ. Solid paraffin was used as the binder, mixing it evenly with the BIQ-adsorbed EG to make a filler, successfully preparing a BIQ-modified EG paste electrode. The electrode has a wide detection range, low detection limit, and good reliability, and it exhibits good selectivity for Cu^2+^ under various interferences. Experimental results show that under optimal conditions, the linear equation for the peak oxidation current with Cu^+^ concentration ranging from 0.05 mg/L to 1.0 mg/L is I = 0.03769 + 0.29997 × c (R^2^ = 0.9989), with a detection limit of 8 μg/L (S/N = 3). The developed method can be successfully applied to the selective detection of Cu^+^ in electrolytic copper foil solutions.

## Figures and Tables

**Table 1:** Determination of electrode for cuprous ions in real samples.

Sample	Analyte	Measured by Spectrophotometry mg/L	Measured by This Method mg/L	Recovery/%	RSD/%
Copper foil electrolyte	Cu^+^	0.5124	0.5236	102.19	1.64
			0.5354	104.48	
			0.5186	101.21	
		0.7505	0.7816	104.14	1.44
			0.7596	101.21	
			0.7683	102.37	

## Data Availability

The original contributions presented in this study are included in the article/Supplementary Material. Further inquiries can be directed to the corresponding authors.

## Supplementary Materials

The following supporting information can be downloaded at: https://www.mdpi.com/article/10.3390/ma19030586/s1, Figure S1: DPV curves of the BIQ-adsorbed EG electrode before and after nitrogen sparging: (a) Before nitrogen sparging.; (b) After nitrogen sparging. Figure S2: DPV curves of electrodes prepared with different EG:paraffin ratios. Figure S3: (A) EIS spectra and (B) fitted Rct values of EG electrodes with different BIQ dosages. Figure S4: DPV curves of electrodes prepared under different conditions: (a) EG electrode without BIQ; (b) BIQ-modified EG electrode with no preconcentration; (c) BIQ-modified EG electrode enriched only in Cu^2+^ solution. Figure S5: FT-IR spectra of (a) Filtered residue; (b) expanded graphite (EG) adsorbed with 2,2′-biquinoline (BIQ). Table S1: BET and pore structure parameters of FG and EG.
