# Supplementary material for: 2,2′-Biquinoline Modified Expanded Graphite Electrode for the Detection of Cuprous Ions in Electrolytic Copper Foil Electrolyte
Source: Materials (Basel). 2026 Feb 3;19(3):586. doi: 10.3390/ma19030586 (PMC12898254; doi:10.3390/ma19030586)
Supplement: Supplementary file 1 [file materials-19-00586-s001.zip › materials-4111702-supplementary.pdf]

Supporting Information

Regarding the sensitivity of the DPV signal to oxygen content, as shown in Figure 1, a comparison of the DPV test signals before and after deoxygenation via nitrogen bubbling revealed that the peak current was 0.3065 μA prior to nitrogen bubbling, and after nitrogen bubbling, the peak current increased to 0.3136 μA, corresponding to a mere increase of 2.3%. This indicates that the signal has low sensitivity to oxygen content. This result is primarily attributed to the chemical protection effect of excess HA, which effectively offsets the oxidative impact of trace dissolved oxygen on Cu^{+} and ensures the stability and accuracy of the detection signal.

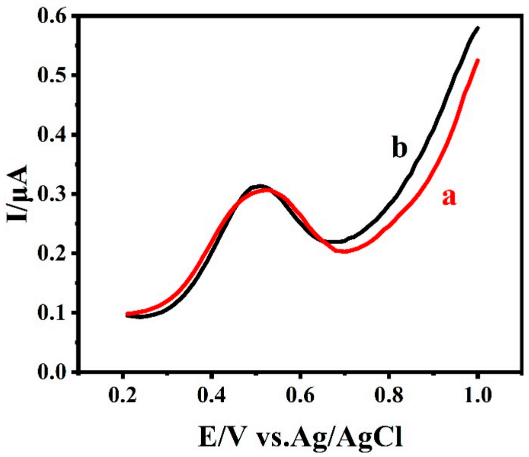

Figure S1. DPV curves of the BIQ-adsorbed EG electrode before and after nitrogen sparging: (a) Before nitrogen sparging. (b) After nitrogen sparging.

We conducted BET analysis on FG and EG separately, with partial derived parameters shown in Table S1. The FG sample has a BET specific surface area of 1.4348 m^2/g and an average pore diameter of 1.46760 nm, exhibiting prominent microporous characteristics. In contrast, the EG sample has a BET specific surface area of 28.7537 m^2/g, which is 20.4 times that of FG, and a single-point total adsorption pore volume of 0.127200 cm^3/g (8 times that of FG), and it is mainly characterized by meso/macroporous volumes.

The differences in pore structures between FG and EG directly determine their performance. The larger BET specific surface area of EG provides approximately 20 times more BIQ adsorption sites; combined with its 8-fold higher total pore volume, the actual BIQ adsorption capacity of EG is significantly enhanced. Additionally, the wider average pore diameter and higher external specific surface area of EG led to a larger diffusion coefficient of BIQ within it. Collectively, these data demonstrate the superior performance of the EG-based electrode.

Table S1. BET and pore structure parameters of FG and EG

Parameter	FG	EG
**BET surface area /m^2g^{-1}**	1.43485	28.7537
**Adsorption average pore diameter (4V/A)/ nm**	1.46760	18.4173

**Total pore volume /cm^{3}g^{-1}**	0.01592	0.12720

We have systematically optimized the content of solid paraffin binder under the premise of keeping the dosage of BIQ independent—specifically, the mass ratio of BIQ to EG was fixed in the experiment, with only the mixing ratio of EG to paraffin adjusted. It was found that when the EG/paraffin ratio exceeded 1:2, excess paraffin would cover the porous structure of EG and the active sites of BIQ, impeding the contact between the electrolyte and active components as well as blocking the charge transfer path between EG particles. Ultimately, this resulted in the failure to detect the characteristic oxidation peak at 0.55 V in DPV measurements.

Within the aforementioned threshold range, we further set five gradient ratios (EG/paraffin = 3:1, 3:2, 3:3, 3:4, 3:5), fabricated five independent electrodes, respectively, and conducted DPV tests (results are shown in Figure S2). A comparison of the peak current responses under different ratios revealed that the electrode achieved the highest characteristic peak current when the EG/paraffin ratio was 3:2. At this ratio, paraffin can not only effectively bind EG and BIQ particles to ensure electrode structural integrity but also avoid excessive coverage of active sites or pore blockage, rendering both charge transfer and complex diffusion in an optimal state.

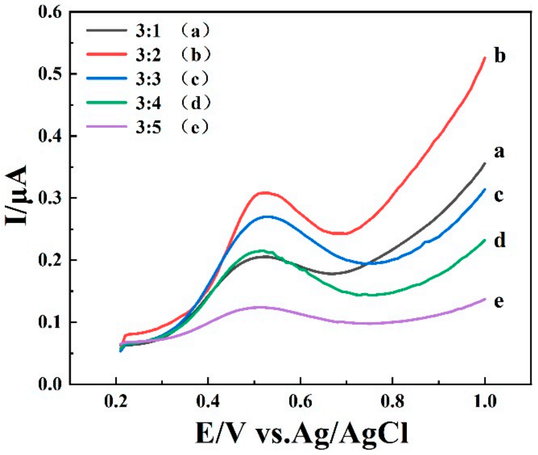

Figure S2. DPV curves of electrodes prepared with different EG:paraffin ratios.

We have supplemented and completed EIS tests of EG electrodes with different BIQ dosages, and obtained Rct data through equivalent circuit fitting, as shown in Figure S3.

The EIS test results indicate that the Rct value of the electrode shows a significant increasing trend with the increase in BIQ dosage: the initial EG electrode without BIQ has an Rct value of approximately 144 Ω; when the BIQ dosage is 1 mg, the Rct value slightly increases to 156 Ω (an 8.3% increase compared to the initial value); as the dosage increases to 5 mg, the Rct value reaches 189 Ω, which is 31.2% higher than that of the initial EG electrode; when the BIQ dosage is further increased to 7 mg, the Rct value surges to 251 Ω, with an increase of up to 74.3% compared to the initial electrode.

The core reason for this variation trend is as follows: an appropriate amount of BIQ can be uniformly modified on the EG surface through π-π stacking. At this time, BIQ mainly binds to the basal plane of EG, without significantly blocking the active edge sites and pore structure

of EG, allowing efficient charge transfer. However, when the BIQ dosage exceeds the threshold of 5 mg, excess BIQ molecules undergo multi-layer adsorption or aggregation on the EG surface, which not only covers the active edge sites of EG but also clogs its porous structure. This blocks the charge transfer paths at the interface between the electrode and the electrolyte, thereby leading to a rapid increase in the Rct value.

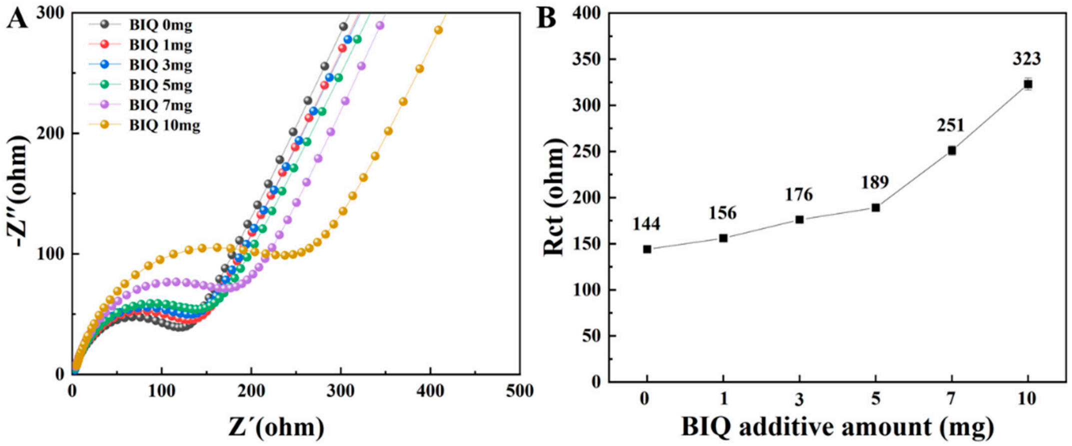

Figure S3. (A) EIS spectra and (B) fitted Rct values of EG electrodes with different BIQ dosages.

To verify whether the characteristic peak at 0.55 V during DPV measurements is dependent on the coexistence of BIQ and Cu(I), we have conducted relevant verification and investigation in our preliminary work, with the results presented in Figure S4., with the corresponding curves as follows: (a) EG electrode without BIQ; (b) BIQ-modified EG electrode without preconcentration; (c) BIQ-modified EG electrode preconcentrated only in Cu^{2+} solution.

No identifiable oxidation peak was observed at 0.55 V in the DPV curves of control experiments (a), (b), and (c), which only exhibited a flat current signal similar to the blank background. Moreover, under the same test conditions, its peak current value was reduced by 76.1% compared with the target system (BIQ-modified EG electrode + Cu^{+} preconcentration). The above results clearly confirm that the generation of the 0.55 V characteristic peak must rely on the synergistic effect of both BIQ and Cu^{+}. Specifically, in the absence of BIQ (Experiment a), the EG electrode lacks specific coordination sites for Cu^{+}, failing to form a stable electrochemical response. In the absence of Cu^{+} (Experiment c), BIQ does not form a specific complex with Cu^{2+}; thus, no target peak is observed. Without preconcentration (Experiment b), there is no Cu^{+} on the electrode surface, resulting in an undetectable signal.

Collectively, the control experiments fully demonstrate that the 0.55 V peak is a specific response to the combined action of BIQ and Cu^{+}.

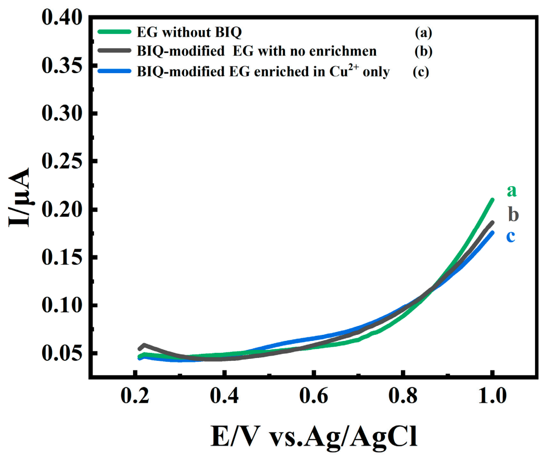

Figure S4. DPV curves of electrodes prepared under different conditions: (a) EG electrode without BIQ; (b) BIQ-modified EG electrode with no preconcentration; (c) BIQ-modified EG electrode enriched only in Cu^{2+} solution.

To clarify the objects of FT-IR analysis and verify surface functionalization, we supplement the following explanation: Figure S5 presents the FT-IR spectra of the EG residue adsorbed with BIQ and the solid BIQ-modified EG material after BIQ adsorption. The characteristic peak positions of the two samples are exactly consistent, with both showing the characteristic peaks of EG and BIQ simultaneously. This result directly confirms that BIQ does not remain only in the solution, but is successfully loaded onto the EG surface through physical adsorption and π-π stacking interactions, indicating that the surface functionalization of the solid BIQ-modified EG material has been clearly achieved.

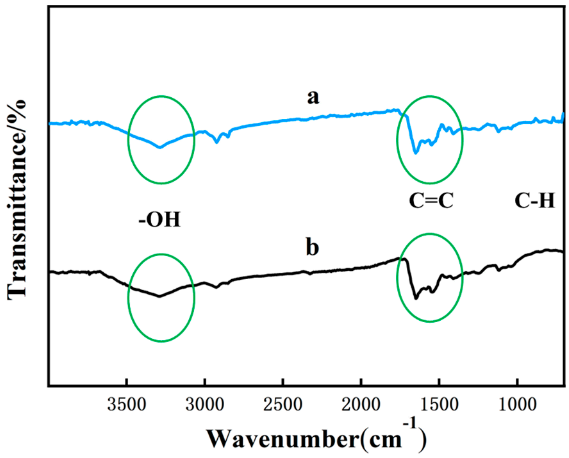

Figure S5. FT-IR spectra of (a) Filtered residue; (b) expanded graphite (EG) adsorbed with 2,2'-biquinoline (BIQ).
